# Declining malaria transmission in rural Amazon: changing epidemiology and challenges to achieve elimination

**DOI:** 10.1186/s12936-016-1326-2

**Published:** 2016-05-10

**Authors:** Sheila Vitor-Silva, André Machado Siqueira, Vanderson de Souza Sampaio, Caterina Guinovart, Roberto Carlos Reyes-Lecca, Gisely Cardoso de Melo, Wuelton Marcelo Monteiro, Hernando A. del Portillo, Pedro Alonso, Quique Bassat, Marcus Vinícius Guimarães Lacerda

**Affiliations:** Fundação de Medicina Tropical Dr. Heitor Vieira Dourado, Av. Pedro Teixeira, 25, Dom Pedro, Manaus, AM 69040-000 Brazil; Universidade do Estado do Amazonas, Av. Pedro Teixeira, 25, Dom Pedro, Manaus, AM 69040-000 Brazil; Instituto Nacional de Infectologia Evandro Chagas, Fundação Oswaldo Cruz, Av. Brasil, 4365, Manguinhos, Rio de Janeiro, RJ 21040-360 Brazil; ISGlobal, Barcelona Ctr. Int. Health Res. (CRESIB), Hospital Clínic–Universitat de Barcelona, Rosselló 132, 4°, 08036 Barcelona, Spain; Secretaria de Vigilância em Saúde, Ministério da Saúde, Lotes 5/6 Bloco F, SAF Sul Trecho 2, Brasília, DF 70070-600 Brazil; Institució Catalana de Recerca i Estudis Avançats (ICREA), Barcelona, Spain; Centro de Investigação em Saúde de Manhiça (CISM), Maputo, Mozambique; Instituto de Pesquisas Leônidas & Maria Deane, Fundação Oswaldo Cruz, Rua Terezina, 476, Adrianópolis, Manaus, AM 69057-070 Brazil

**Keywords:** Malaria, *Plasmodium vivax*, *Plasmodium falciparum*, Elimination, Health Surveillance, Amazon

## Abstract

**Background:**

In recent years, considerable success in reducing its incidence has been achieved in Brazil, leading to a relative increase in the proportion of cases caused by *Plasmodium vivax*, considered a harder-to-eliminate parasite. This study aim is to describe the transmission dynamics and associated risk factors in a rural settlement area in the Western Brazilian Amazon.

**Methods:**

A prospective cohort was established in a rural settlement area for 3 years. Follow-up included continuous passive case detection and monthly active case detection for a period of 6 months. Demographic, clinical and transmission control practices data were collected. Malaria diagnosis was performed through thick blood smear. Univariable and multivariable analyses of factors associated with malaria incidence were performed using negative binomial regression models. Factors associated with recurrence of *P. vivax* and *Plasmodium falciparum* malaria within 90 days of a previous episode were analysed using univariable and multivariable Cox-Proportional Hazard models.

**Results:**

Malaria prevalence decreased from 7 % at the study beginning to 0.6 % at month 24, with *P. vivax* predominating and *P. falciparum* disappearing after 1 year of follow-up. Malaria incidence was significantly higher in the dry season [IRR (95 % CI) 1.4 (1.1–1.6); p < 0.001)]. Use of ITN was associated to malaria protection in the localities [IRR (95 % CI) 0.7 (0.6–0.8); p = 0.001)]. A recurrent *P. vivax* episode within 90 days was observed in 29.4 % of individuals after an initial diagnosis. A previous *P. vivax* [IRR (95 % CI) 2.3 (1.3–4.0); p = 0.006)] or mixed *P. vivax* + *P. falciparum* [IRR (95 % CI) 2.9 (1.5–5.7); p = 0.002)] infections were significantly associated to a vivax malaria episode within 90 days of follow-up.

**Conclusions:**

In an area of *P. falciparum* and *P. vivax* co-endemicity, a virtual disappearance of *P. falciparum* was observed with *P. vivax* increasing its relative contribution, with a large proportion of recurring episodes. This finding reinforces the perception of *P. falciparum* being more responsive to early diagnosis and treatment and ITN use and the contribution of relapsing *P. vivax* to maintain this species’ transmission. In areas of *P. vivax* endemicity, antihypnozoite treatment effectiveness assessment in different transmission intensity may be a fundamental activity for malaria control and elimination.

**Electronic supplementary material:**

The online version of this article (doi:10.1186/s12936-016-1326-2) contains supplementary material, which is available to authorized users.

## Background

Malaria is a preventable, diagnosable and treatable disease. With innovation and roll out of interventions there are fewer people dying from malaria now than in any other historical period. During 2015, there were an estimated 214 million cases of malaria and an estimated 438,000 deaths (range 236,000–635,000) globally [1]. Following declarations at the malaria Forum in 2007 convened by the Bill & Melinda Gates Foundation (BMGF), and later endorsed by the World Health Organization (WHO), the international community has embraced the goal for scaling up malaria control to regional elimination and the ultimate goal of global eradication. Today, there is on-going malaria transmission in 97 countries; twenty-six of these countries are in the ‘pre-elimination’, ‘elimination’ or ‘prevention of re-introduction’ stages and four countries have been WHO certified malaria-free [2]. Based on reported data, in the Region of the Americas, reductions in incidence of >75 % in malaria cases were reported in 13 out of 21 countries with ongoing transmission between 2000 and 2012. Although Brazil has achieved an important reduction of malaria transmission, it still accounts for 41.7 % of malaria in the Americas [3].

After the World Malaria Eradication Campaign, implemented in Brazil in the 1960s, the most successful achievement was malaria elimination outside the Amazon. Since then, transmission has been almost entirely restricted to the Amazon, with periods of epidemics occurring in parallel with increasing migration to the region, in part enhanced by development projects subsidized by the government, such as building of roads and the creation of a tax-free industrial zone in Manaus [4]. From 1992 (the Amsterdam Conference) to 2000, Brazil has achieved the goal of reducing substantially the percentage of *Plasmodium falciparum* infections and, therefore, the number of hospitalizations and deaths [5]. Since 2000, an expansion in the financing and coverage of the National Malaria Control Programme (NMCP) has led to a wide-scale reduction in malaria incidence and mortality, with considerable shrinkage of the malaria transmission area towards the Western Amazon region, fostering the expectations for planning for elimination in the near future [6].

In the context of malaria elimination, *Plasmodium vivax* has been receiving increasing attention due to the perception of a greater difficulty to achieve its control as compared to *P. falciparum* [7], with estimated 2.48 billion people living at risk of infection [8]. Relapses arising from hypnozoites in the liver are a common feature of *P. vivax* malaria. These can occur weeks to years after the initial infection and place further health burdens on those affected. Studies from Brazil showed relapse rates ranging from 14 to 40 % [7–9], thus providing an important source of reinfection [10, 11] and hampering malaria control. Relapse prevention requires chemotherapy with primaquine that targets the latent liver stages of *P. vivax* [12].

Detailed community-based data, including age patterns, incidences and seasonality, are needed not only for a better understanding of the problem and possibilities of control and elimination, but also to have baseline data to substantiate future interventions, such as vector control, mass drug administration or eventually vaccines. This work aimed to describe the epidemiology of malaria in an endemic area in the surrounding areas of Manaus (Brazil), in the Western Brazilian Amazon, including the risk factors for incidence and recurrent parasitaemia in this endemic area.

## Methods

### Study sites

The Municipality of Careiro is located in the central region of the Amazonas State (Western Brazilian Amazon) (03°06′ S; 60°01′ W), 112 km from the capital of the state, Manaus. The estimated population in 2010 was 32,734 people, mainly living in rural areas (71.2 %). The main economical activity is related to agriculture (cassava, fruits, vegetables, rice, and sugar cane), cattle breeding, fish-farming, and forestry. These activities have resulted in a decrease in vegetation cover and habitat fragmentation. The remaining vegetal cover is primarily composed of dense macrothermic ombrophilous forest. The climate according to Köppen classification is Af (super-humid equatorial), with a mean pluviometric precipitations above 2000 mm *per annum*, average temperatures ranging from 26 to 30° C, and relative humidity between 85 and 90 %. A rainy season occurs from November to April, with an average monthly rainfall of 290 mm in this season and a peak in February (390 mm); a dry season occurs from May to October, with an average monthly rainfall of 110 mm in this season and lowest values in September (45 mm). Mean temperature in the period values were 27.6 and 26.9 °C in the dry and rainy seasons, respectively [13].

**Fig. 1:**
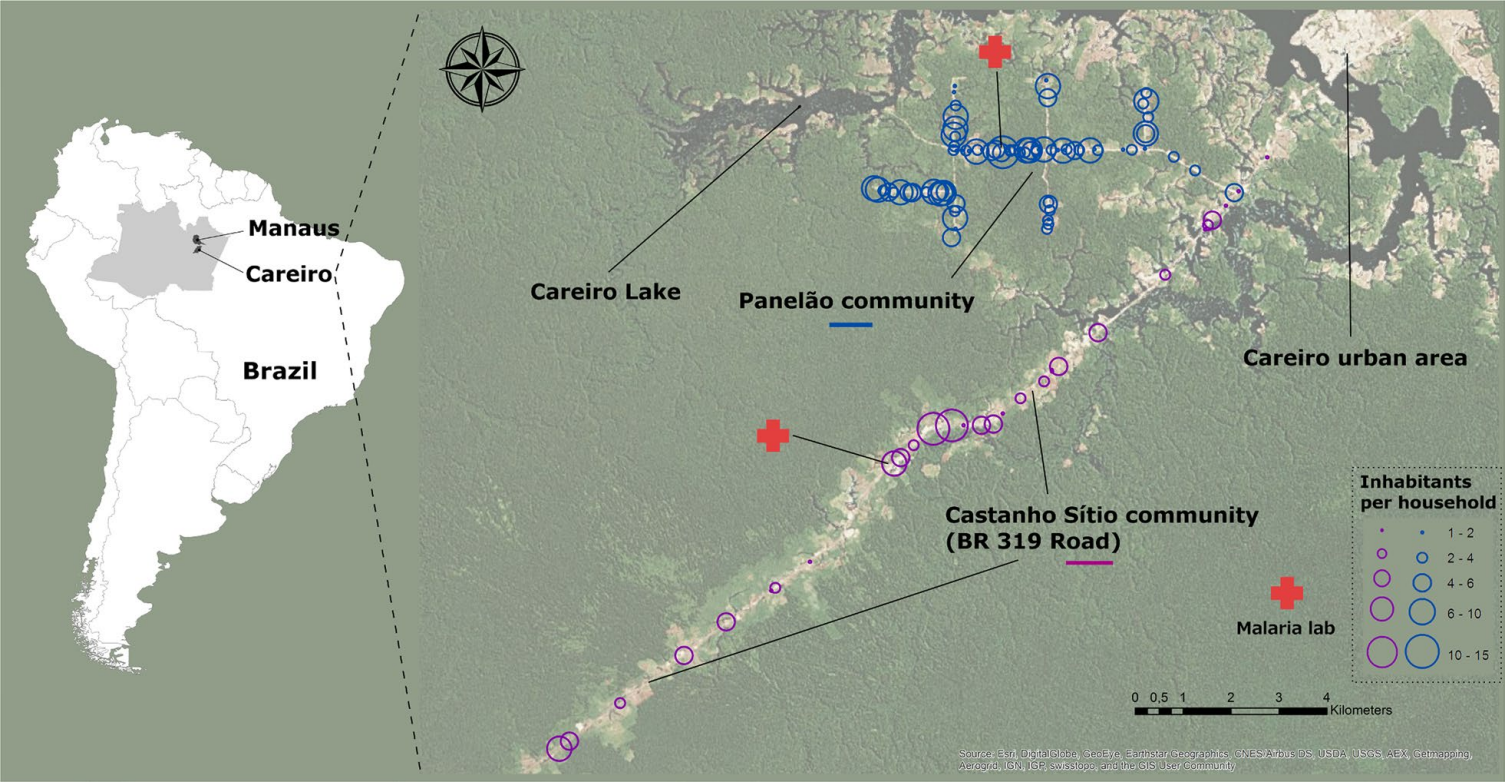
Area of study, with the houses and malaria clinics location in the two communities

Two areas devoted to agriculture were chosen (Panelão Community, a rural settlement, and Castanho Sítio, consisting of ranches along the Manaus-Porto Velho road), with a total population of 821 persons (census performed immediately before the beginning of the study). The two areas were established in the 90 s and present a profile of relative low migration and similar environmental, socio-economical and demographical features. These characteristics, linked to the easy access from Manaus, determined in selecting these study areas. Official surveillance shows that malaria is moderately transmitted in the communities, with notable seasonality, which is characterized by higher transmission intensity in the dry season, when temporary breeding sites form on the river margins [4]. Before 2004, *P. vivax* and *P. falciparum* presented similar incidence rates in the area; from 2004 on, *P. vivax* predominates (Additional file 1: Figure S1). Breeding sites for anophelines are common in the surrounding areas and *Anopheles darlingi* is the major malaria vector in the communities [14]. The study area is presented in Fig. 1.

The study villages were served with community microscopists of local malaria diagnosis and treatment units (Fig. 1). As a national policy, a blood smear is collected for all febrile cases suspicious of malaria and information is registered using a standardized malaria surveillance system. Blood slides are read immediately and if the slide is positive anti-malarial treatment is provided by the health worker to the patient.

### Data retrieval

A prospective cohort study was carried out for 24 months. Before the start of the study (June 2008), the whole population of the areas was submitted to a preliminary census. At this moment the community was sensitized, informed about the study and invited to participate. Follow-up of study participants was done through cross-sectional surveys and passive case detection at the health posts for the entire duration of the study. In total the team conducted one visit for census and sensitization and cross-sectional surveys every 6 months.

During the five cross-sectional surveys a thick blood smear was performed for all individuals within the cohort, with or without malaria symptoms, through home visiting. At this time if one of the individuals in the cohort were not found, only a new visit was scheduled. Moreover, new residents identified during the cross-sectional surveys were invited to enter the cohort. The first (August 2008; T0), the third (August 2009; T12) and the fifth (August 2010; T24) cross-sectional surveys were performed during the dry season and the second (February 2009; T6) and the fourth (February 2010; T18) during the rainy season.

For malaria patients reported in the study, first-line treatment recommended by the Brazilian Ministry of Health to *P. vivax* malaria (chloroquine for 3 days and primaquine for 7 days) and for *P. falciparum* (artemether plus lumefantrine for 3 days and primaquine on the 1 day) were prescribed [12].

### Active follow-up

Visits in the cross-sectional surveys included a clinical history, a physical exam and blood sampling. A finger prick blood sample (≈200 µL) was collected to prepare two blood smears for detection of *Plasmodium* infection. Information was recorded on standardized forms. Blood slides were read at the health post within 24 h and both symptomatic and asymptomatic patients with a positive thick blood smear detected during the cross-sectionals were treated by the study team and/or referred to the local hospital.

### Passive case detection

For the purpose of this study, blood was collected to prepare two slides from all patients presenting with fever or symptoms compatible with malaria, for individuals assisted at the diagnosis and treatment units or at the home visits. Following the normal routine of the health service of the communities, one blood slide was read immediately and if the slide was positive anti-malarial treatment was provided by the health worker to the patient. Information was registered in a CRF by the community health workers and microscopists. The second slide collected was labelled with the study code and taken to Manaus, where it was reviewed by two microscopists to confirm diagnosis and for parasite density determination. Malaria surveillance standardized forms included demographic data, clinical characteristics of the patient and parasitaemia results. These forms were sent to Manaus on a monthly basis, where they were entered into databases by two independent data clerks in an OpenClinica^®^ online platform.

### Malaria diagnosis and definitions

For all study participants, thick blood smears were prepared as recommended by the Walker technique [15] and evaluated by a local microscopist. The slides were then sent to Manaus and reviewed by an experienced microscopist, who confirmed diagnosis. Parasitological results were confirmed by RT-PCR [16]. A calibrated clinical examination was made in all subjects submitted to thick blood smears examination in the health post. Data on the presence of fever, headache, chills, myalgia, weakness, sweating, abdominal pain, anorexia, nausea, cough, diarrhoea, vomiting, nasal flaring (only in children <10 years), crackles, wheezing or ronchi, pallor, dehydration, oedema and jaundice were recorded. At this moment, patients were asked also if they took anti-malarials in the last 30 days.

### Statistical analysis

The incidence rates of *Plasmodium*-specific malaria were calculated by dividing the respective number of episodes by the total person-years at risk in the moment, obtaining overall and group-specific rates and respective survival curves using Kaplan–Meier method. For describing the monthly incidence of malaria and positivity rates, all slides performed at each calendar month were combined to allow the calculation of monthly incidence of cases per species and the slide positivity rate, obtained by dividing the malaria positive cases by the total of fever cases that had a blood slide performed. Confidence intervals were obtained using the quadratic approximation to the Poisson log likelihood for the log-rate parameter as described in the Stata software manual.

Malaria distribution was analysed with a kernel density estimator using the spatial analyst tool from ArcGIS 10.1 (ESRI, California, USA). Incidence of disease per dwelling was used as population field. Output cell size and search radius were set to 0.00079 and 0.0066, respectively.

Univariate and multivariate analyses of factors associated with the incidence of malaria (specific and per-species) were performed by comparing the parasite-specific incidence rates of malaria episodes between groups using a negative binomial regression model with robust standard error to account for clustering by individual. The investigation of factors associated with recurrence of *P. vivax* and *P. falciparum* malaria within 90 days of a previous episode was analysed using univariable and multivariable Cox-Proportional Hazard models, with robust standard errors.

### Ethics approval

Human surveys were approved by the National Ethics Review Committee (protocol number 15197/2008, and the Ethics Committee of the Hospital Clínic, Barcelona, Spain. Informed consent was obtained from each participant. All malaria cases detected in the longitudinal or cross-sectional studies were treated according to the Brazilian Ministry of Health guidelines. Patients with any clinical complication were referred to the Careiro Hospital.

## Results

### Study population

**Fig. 2:**
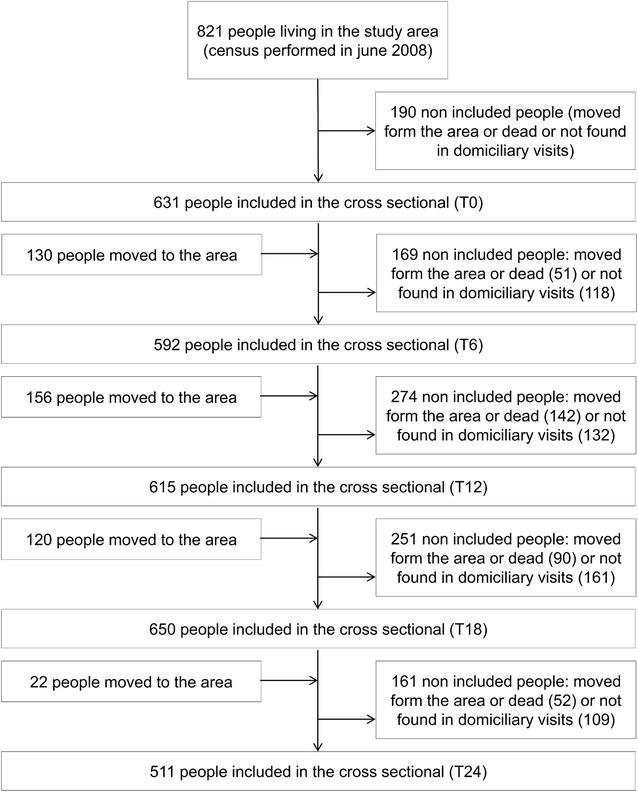
Flow chart of inclusion along the prospective cohort

The census performed before the beginning of the study (June 2008) showed 821 people living in the study area. The first cross-sectional (August 2008; T0) included 631 people, the second (February 2009; T6) included 592 people, the third (August 2009; T12) included 615 people, the fourth (February 2010; T18) included 650 people and the fifth (August 2010; T24) included 511 people (Fig. 2). Subjects included were mostly males (57 %). The most frequent age groups were 15–35 years old (28 %) and 5–15 years old (27 %). Ethnically, people are mostly admixed (98.7 %). In relation to education, subjects had mostly elementary school (68 %). Farmer/fisher was the most common occupation in the area (39 %) (Table 1). A total of 3131 episodes of fever were assessed in the period, respectively 1839 in year 1, 956 in year 2 and 336 in year 3.

**Table 1:** Basic demographic information of the total of subjects included in the prospective study

Variable	Results (n and %)
*Sex*	
Male	664 (57.0 %)
Female	502 (43.0 %)
*Age (years)*	
<5	152 (13.0 %)
5–15	309 (26.5 %)
15–35	321 (27.5 %)
35–50	185 (16.0 %)
>50	199 (17.0 %)
*Ethnics*	
White	9 (0.8 %)
Admixed^a^	1151 (98.7 %)
Black	5 (0.4 %)
Asian	1 (0.1 %)
*Education*	
Illiterate	259 (22.2 %)
Elementary school	792 (68 %)
Middle school	105 (9 %)
Higher school	10 (0.8 %)
*Occupation*	
Farmer/fisher	457 (39.2 %)
Urban employee	26 (2.2 %)
Housewife	81 (6.9 %)
Student	375 (32.2 %)
Retired	40 (3.4 %)
Unemployed	10 (0.9 %)
Other	177 (15.2 %)

^a^The term admixed was used as the traduction for the Portuguese term “*pardo*”, used officially by the Brazilian Institute of Geography and Statistics (IBGE), referring to a wide range of skin colours and backgrounds. They are typically a mixture of White Brazilian, Afro-Brazilian and Native Brazilian

### Malaria burden

**Fig. 3:**
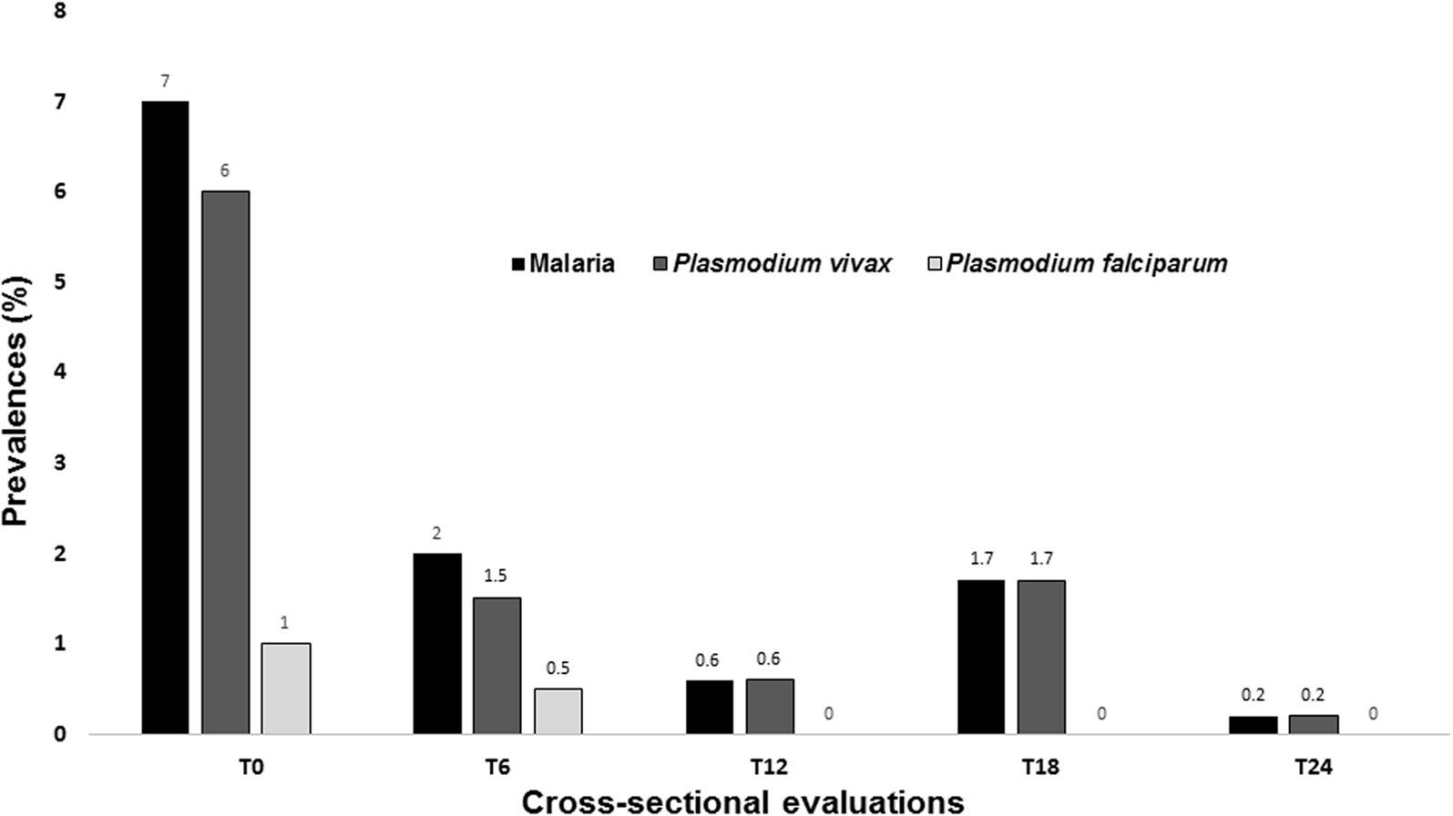
Malaria prevalence by species in the five cross-sectional surveys performed in the study obtained by active case detection

Prevalence of malaria was higher at T0 (7.0 %), dropping to 1.5 % at T6. From T0 to T24, malaria prevalence presented a decreasing trend, with a point prevalence of 0.2 % at T24. *Plasmodium falciparum* infections were recorded only at T0 (1 %) and T6 (0.5 %). *Plasmodium vivax* was responsible by 87.1 % of all infections observed in the cross-sectional evaluations (Fig. 3).

**Fig. 4:**
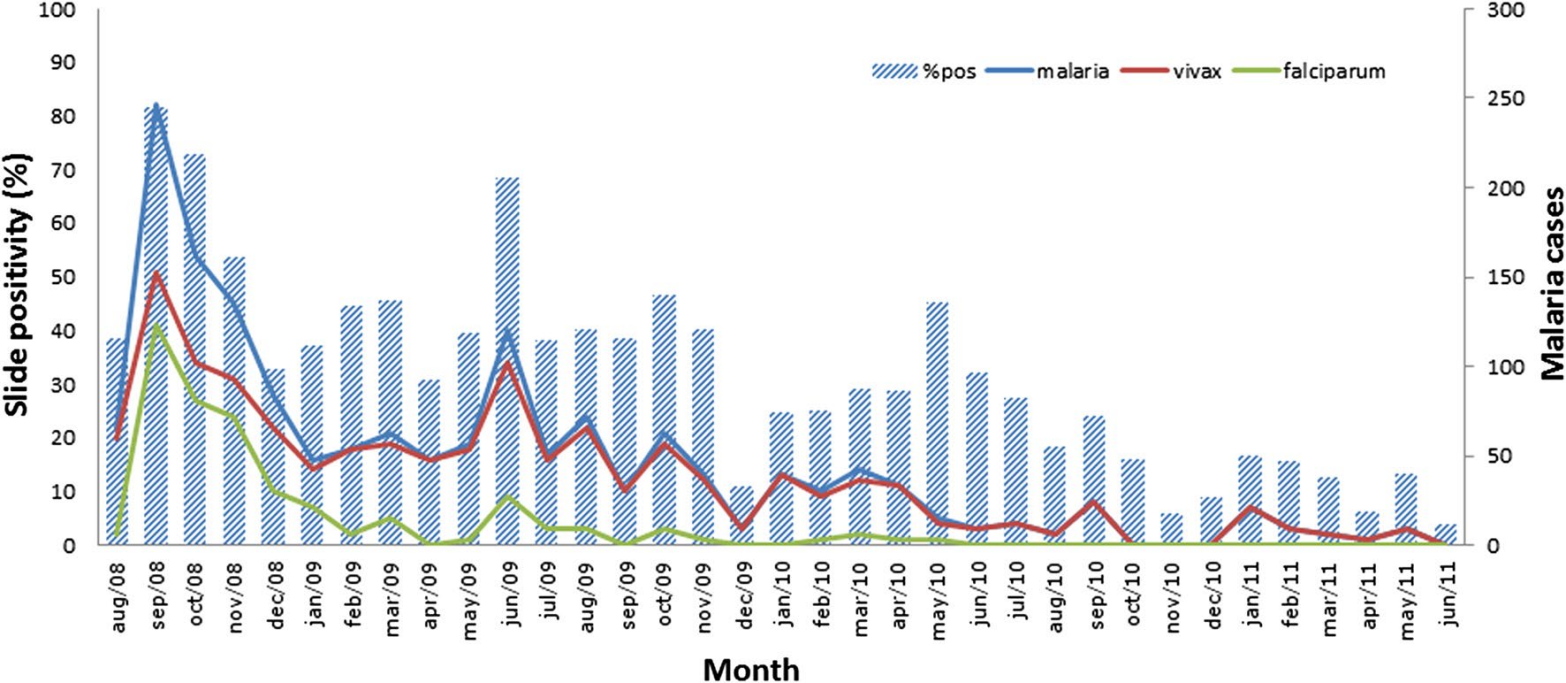
Malaria incidence per species (*lines*) and slide positivity rates trends per calendar month obtained from the passive case detection of fever cases during the period of study

In total, 550 malaria episodes were registered in the period, 408 in the first year, 120 in the second and 22 in the third, yielding rates of 847, 150 and 41 episodes/1000-person-year, respectively. The number of episodes per individual in a given year varied from one to seven (maximum of three for *P. falciparum*), and among individuals presenting vivax malaria in the follow-up, 20.4 % had more than one episode. In overall, *P. vivax* malaria incidence decreased from 22.1 to 7.9 % and *P. falciparum* malaria incidence decreased from 14.1 to 0.6 % in the period. The incidence of recurrent vivax malaria also decreased over the study period, from 3.9 % in 2008 to 0.7 % in 2010. Among *P. falciparum* patients, 11.1 % developed more than one episode. Figure 4 shows the decreasing patterns of malaria incidence per species over the period. As transmission intensity decreased, the proportion of febrile cases dropped considerably. In the beginning of the study, more than 70 % of febrile cases had positive slides, while in the end of the period, slide positivity rate was to less than 10 %.

Malaria cases predominated in the groups aged between 10 and 60 years-old. This profile was also observed also for *P. vivax* and *P. falciparum* malaria. A proportion of 8.5 % of the malaria cases were recorded from children under 5 years of age.

**Fig. 5:**
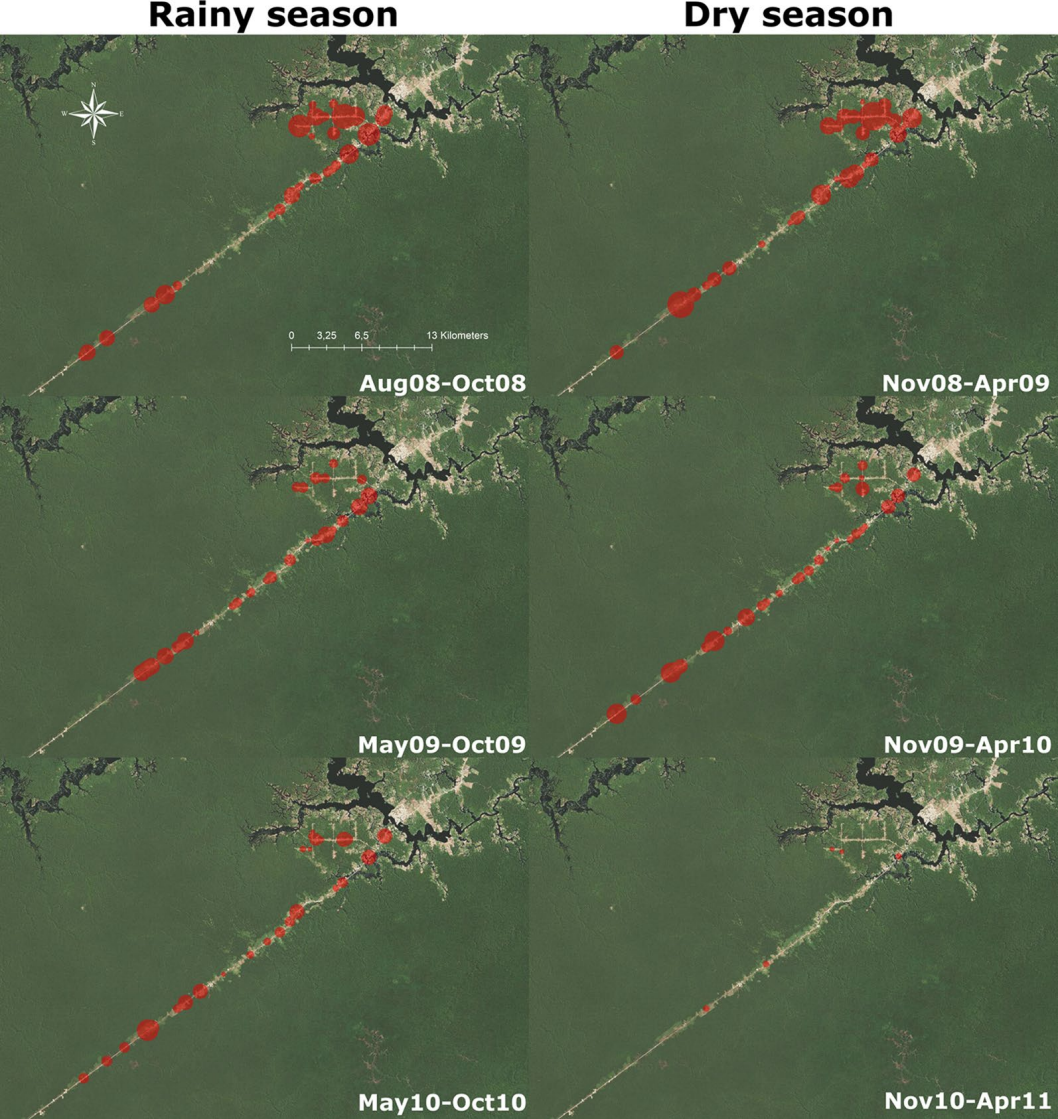
Spatial and temporal variation of malaria incidence in the study area

Spatial distribution shows that malaria cases were unevenly distributed in the study area, with higher incidences in the Panelão Community, in the major road and in three minor sideroads. In the Castanho Sítio Community, malaria cases were more sparsely distributed probably reflecting this area configuration (Fig. 5). Temporally it was possible to observe changes in the “hotspots” locations, demonstrating the dynamic characteristic of transmission in the region.

### Malaria control activities

**Fig. 6:**
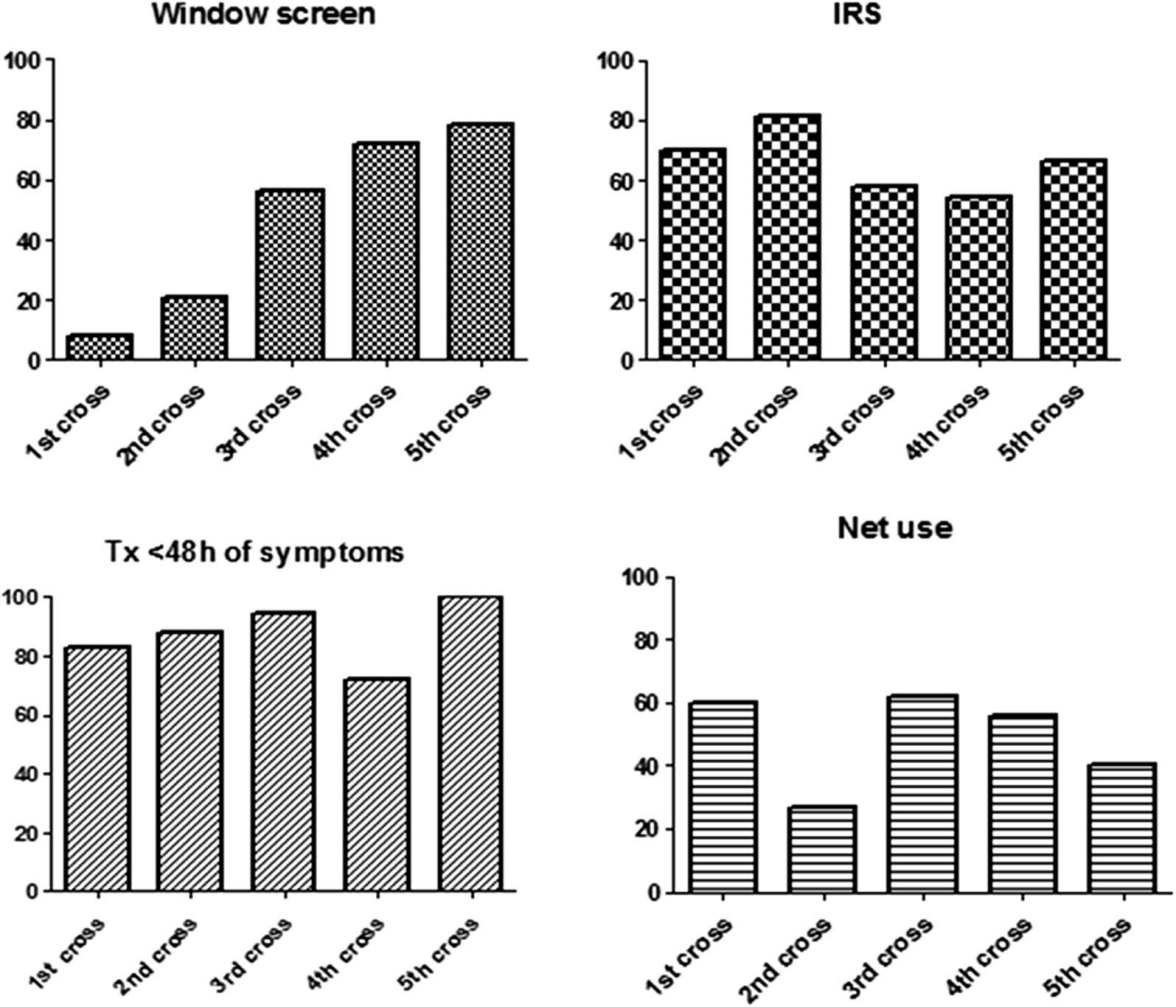
Operational measures for malaria control coverage in the study area over the period of study

Coverage of malaria control measures is presented in Fig. 6. Window screening use increased from 8 % in T0 to 78 % of the houses in T24. In the beginning of the follow-up (T0), 188 (70 %) of the houses had been submitted to indoor residual spraying (IRS; cypermethrin) in the preceding 6 months. IRS coverage slightly increased in the period, from 54 % of the houses at T18 to 81 % at T6. A total of 378 (60 %) of the subjects referred using bed nets in the previous night. In the same way, use of bed net ranged from 27 % at T6 to 62 % at T12. However, insecticide-treated bed nets started to be used only at the third cross-sectional (T12). In general, patients searched for health care within <48 h of symptoms (80.2 %). Only 3 % of malaria cases were diagnosed after 7 days after symptoms had started. Percentage of cases diagnosed within 48 h of symptoms ranged from 81.5 % at T0 to 97.3 % at T24.

### Incidence rate and hazard ratios for malaria

The risk of malaria infection was significantly higher in the dry season [IRR (95 % CI) 1.4 (1.1–1.6); p < 0.001)] and wherever IRS had been used in the last 6 months [IRR (95 % CI) 1.4 (1.1–1.8); p = 0.002)]. Use of ITN was associated to malaria protection in the localities [IRR (95 % CI) 0.7 (0.6–0.8); p = 0.001)]. Gender, age and socioeconomic status were not related to malaria incidence. Stratification by malaria species showed similar results (Tables 2 and 3). However, ITN use seemed to have a more protective effect on *P. falciparum* malaria [IRR (95 % CI) 0.5 (0.3–0.7); p < 0.001)] in comparison with *P. vivax* malaria [IRR (95 % CI) 0.7 (0.6–0.8); p = 0.001)].

**Table 2:** Incidence rate and hazard ratios for *Plasmodium vivax* episodes according to exposure status

	N episodes	Person-years of follow-up	Rate (episodes/100-PY) (95 % CI)	Univariable IRR (95 % CI)	*P* value	Multivariable IRR (95 % CI)	P value
*Gender*							
Female	279	802	34.8 (31.0–39.1)	1	0.151	1	0.204
Male	377	998	37.8 (34.2–41.8)	1.2 (0.9–1.5)		1.2 (0.9–1.5)	
*Age* (*years*)							
0–10	194	451	43.0 (37.3–49.5)	1		1	
10–20	143	436	32.8 (27.8–38.6)	0.7 (0.6–1.0)	0.059	0.7 (0.5–0.9)	0.038
20–40	163	429	38.0 (32.6–44.3)	0.9 (0.7–1.3)	0.709	1.0 (0.7–1.3)	0.954
40–60	114	328	34.7 (28.9–41.7)	0.9 (0.6–1.2)	0.461	0.9 (0.6–1.3)	0.524
≥60	42	153	27.3 (20.2–36.9)	0.7 (0.5–1.2)	0.195	0.8 (0.5–1.2)	0.229
*Socio*-*economic status*							
Lowest quintile	258	702	36.7 (32.5–41.5)	1.0 (0.8–1.2)	0.798	0.9 (0.7–1.1)	0.422
2nd–4th quintiles	284	707	40.2 (35.8–45.1)	1		1	
Highest quintile	114	320	35.6 (29.6–42.7)	1.0 (0.7–1.3)	0.825	0.9 (0.7–1.2)	
*Season*							
Rainy	238	750	31.7 (27.9–36.0)	1	0.007	1	<0.001
Dry	418	1049	39.9 (36.2–43.9)	1.2 (1.1–1.5)		1.4 (1.1–1.6)	
*Use of ITN*							
No	362	857	42.3 (38.1–46.8)	1	0.199	1	0.001
Yes	294	943	31.1 (27.8–35.0)	0.9 (0.7–1.1)		0.7 (0.6–0.8)	
*IRS in last 6* *months*							
No	149	530	28.1 (23.9–33.0)	1	0.015	1	0.002
Yes	507	1200	42.3 (38.7–46.1)	1.4 (1.1–1.8)		1.4 (1.1–1.8)

**Table 3:** Incidence rate and hazard ratios of *Plasmodium falciparum* episodes according to exposure status

	N episodes	Person-years of follow-up	Rate (episodes/100-PY) (95 % CI)	Univariable IRR (95 % CI)	P value	Multivariable IRR (95 % CI)	P value
*Gender*							
Female	79	802	9.9 (7.9–12.3)	1		1	0.249
Male	118	998	11.8 (9.9–14.2)	1.2 (0.9–1.8)	0.175	1.2 (0.9–1.8)	
*Age* (*years*)							
0–10	51	451	11.3 (8.6–14.9)	1		1	
10–20	56	436	12.8 (9.9–16.7)	1.1 (0.6–1.7)	0.847	0.9 (0.6–1.6)	0.833
20–40	49	429	11.4 (8.6–15.1)	1.1 (0.7–1.8)	0.756	1.1 (0.7–1.8)	0.746
40–60	28	328	8.5 (6.0–12.3)	0.8 (0.4–1.4)	0.433	0.8 (0.4–1.5)	0.489
≥60	13	153	8.4 (4.9–14.5)	0.8 (0.4–1.9)	0.648	0.8 (0.4–1.9)	0.621
*Socio*-*economic status*							
Lowest quintile	76	702	10.9 (8.6–13.5)	0.9 (0.6–1.3)	0.592	0.9 (0.7–1.4)	0.821
2nd–4th quintiles	92	707	13.0 (10.6–16.0)	1		1	
Highest quintile	29	320	9.1 (6.3–13.0)	0.8 (0.5–1.4)	0.415	0.8 (0.5–1.3)	0.397
*Season*							
Rainy	56	750	7.5 (5.7–9.7)	1	<0.001	1	<0.001
Dry	141	1049	13.4 (11.4–15.9)	1.7 (1.3–2.3)		1.7 (1.3–2.3)	
*Use of ITN*							
No	138	857	16.1 (13.6–19.0)	1	<0.001	1	<0.001
Yes	59	943	6.3 (4.8–8.1)	0.5 (0.3–0.7)		0.5 (0.3–0.7)	
*IRS in last 6* *months*							
No	31	530	5.8 (4.1–8.3)	1	0.001	1	<0.001
Yes	166	1200	13.8 (11.9–16.1)	2.1 (1.3–3.4)		1.9 (1.2–3.3)	

### Risk factors for recurrent malaria episodes

**Fig. 7:**
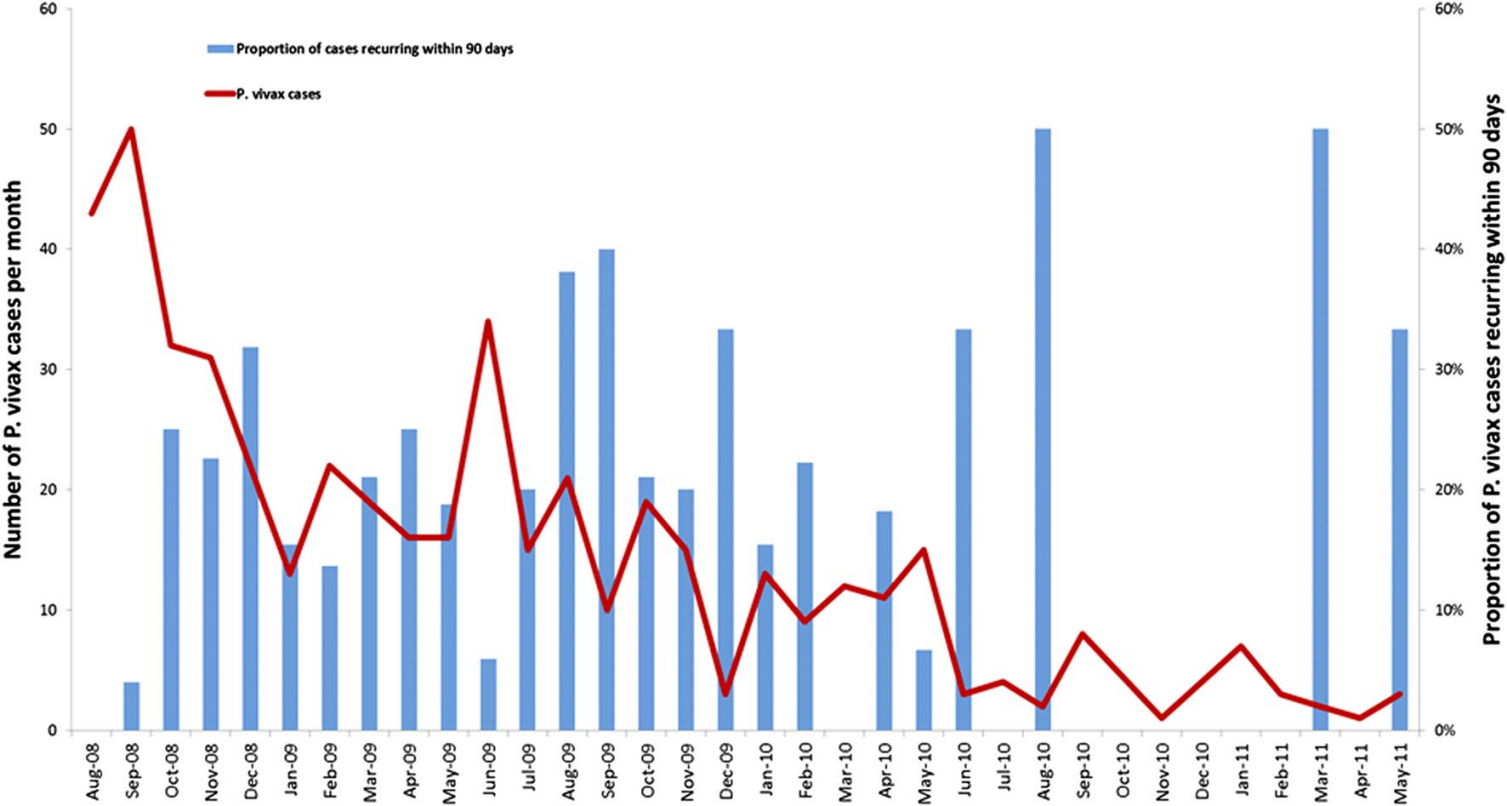
Malaria incidence and recurrent parasitaemias in 90 days per month during the period of study

Proportion of recurrent malaria episodes by *P. vivax* within 90 days of a previous episode was 189/642 (29.44 %) (Fig. 7). Recurrences were observed mostly between the 28 and 365 days after the first vivax malaria episode (81.3 %), and particularly in the 121–365 days time-interval (32.2 %). Early recurrences (until 27 days) were observed in 7.6 %. Late recurrences (≥365 days) were observed in 11.1 %.

**Fig. 8:**
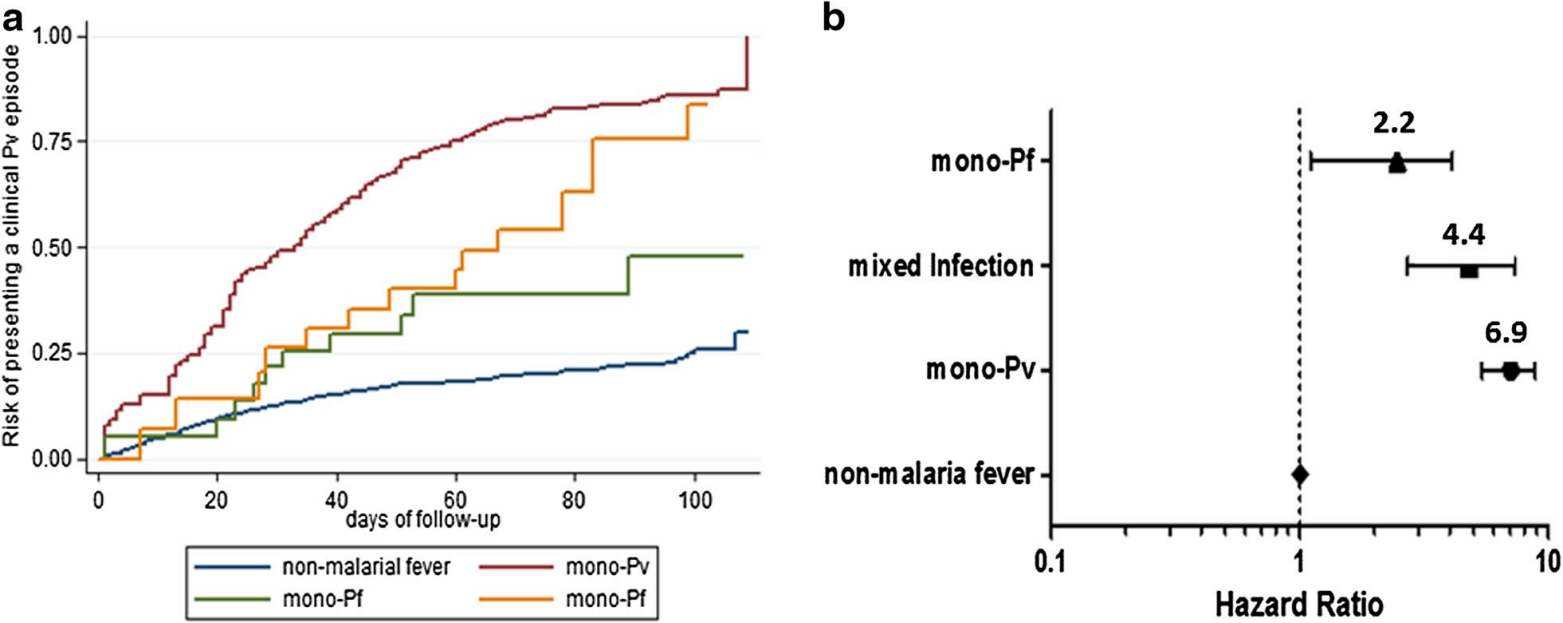
Risk of presenting *P. vivax* recurrence following a malaria episode by either *P. vivax*, *P. falciparum* or mixed infection. **a** Survival analysis of the time for the first malarial episode, showing the time elapsed from a previous non-malarial fever, *P. vivax*, *P. falciparum* or mixed infection until a *P. vivax* clinical episode. **b** Hazard ratios for presenting a *P. vivax* clinical episode to last 120 days, for non-malaria fever, *P. vivax*, *P. falciparum* or mixed infection

A previous *P. vivax* [IRR (95 % CI) 2.3 (1.3–4.0); p = 0.006)] or mixed *P. vivax* + *P. falciparum* [IRR (95 % CI) 2.9 (1.5–5.7); p = 0.002)] infections were significantly associated to a vivax malaria episode within 90 days of follow-up. Recurrent *P. vivax* infections were also significantly higher in the dry season [IRR (95 % CI) 2.5 (1.8–3.5); p = 0.001)] (Table 4). Kaplan–Meier estimates confirmed a higher risk of recurrences after a history of *P. vivax*, mixed *P. vivax* + *P. falciparum* and *P. falciparum* infections (Fig. 8). Risk of *P. vivax* recurrence was not associated to to bed net use (Additional file 2: Figure S2).

**Table 4:** Hazard ratio of a *Plasmodium vivax* infection following a previous malaria episode within 90 days of follow-up

	Rate *P. vivax* episode/100-person-months	Crude HR (95 % CI)	P value	Adjusted HR (95 % CI)	P value
*Initial infection*					
*P. falciparum*	5.3 (3.2–8.7)	1		1	
*P. vivax*	11.4 (9.8–12.2)	2.1 (1.2–3.7)	0.009	2.3 (1.3–4.0)	0.006
Mixed Infection	14.2 (9.4–21.6)	2.7 (1.4–5.1)	0.003	2.9 (1.5–5.7)	0.002
*Gender*					
Female	10.5 (8.5–13.0)	1		1	0.983
Male	10.8 (9.0–12.9)	1.0 (0.8–1.3)	0.870	1.0 (0.8–1.3)	
*Age* (*years*)					
0–10	10.0 (7.7–13.0)	1		1	
10–20	10.1 (7.5–13.7)	1.0 (0.7–1.6)	0.865	1.1 (0.7–1.5)	0.809
20–40	11.1 (8.5–14.5)	1.1 (0.8–1.6)	0.557	1.2 (0.8–1.8)	0.284
40–60	12.3 (9.0–16.9)	1.2 (0.8–1.8)	0.268	1.3 (0.9–2.0)	0.126
≥60	9.6 (5.4–16.8)	0.9 (0.5–1.7)	0.885	1.0 (0.5–1.7)	0.933
*Residence area*					
Road ranches	8.7 (6.5–11.7)	1		1	0.091
Rural settlement	11.4 (9.8–13.3)	1.3 (0.9–1.8)	0.108	1.3 (1.0–1.9)	
*Season*					
Rainy	5.9 (4.4–8.0)	1		1	<0.001
Dry	13.6 (11.7–15.9)	2.4 (1.7–3.3)	<0.001	2.5 (1.8–3.5)	
*Use of ITN*					
No	11.0 (9.2–13.2)	1		1	0.284
Yes	10.3 (8.3–12.7)	0.9 (0.7–1.2)	0.564	0.9 (0.7–1.1)

## Discussion

This study was able to comprehensively characterize the epidemiology of malaria in an area coendemic for *P. falciparum* and *P. vivax*, and in a context of a marked decrease incidence between the beginning and the end of study activities. This confirms *P. vivax* as a more resilient species, with the virtual disappearance of *P. falciparum,* a reducing proportion of malaria-attributed fever and an increasing clustering of malaria episodes with reduced incidence. While *P. falciparum* and *P. vivax* responded each for around 50 % of cases in Brazil in 1988 this situation started changing in the 1990s, leading to the current situation in which *P. vivax* has become the predominant species in recent years (only 16.3 % of cases being due to *P. falciparum*) [4, 5, 17]. This shift has been attributed to a number of factors, namely the expansion of the diagnosis and treatment network, making opportune treatment accessible even in remote areas of the Amazon as part of the universal health system; the use of integrated vector management strategies; and, importantly, the support from political leaders in periods of epidemics [5].

In Brazil, current methods for vector control are based primarily on the use of long-lasting insecticide-treated nets (ITN) and indoor residual spraying (IRS), but there is no specific and systematic evaluation on the impact that these measures have on suppressing anopheline populations and reducing levels of malaria parasite transmission in recent agrarian reform projects in the Amazon [14]. In this study, use of ITN but not IRS was associated to malaria protection, higher for *P. falciparum* (~50 %) when comparing to *P. vivax* (~30 %). *Plasmodium vivax* biological features may have contributed to lower vector control effectiveness in this group. Additionally, among anopheline collections in these communities, there was a predominance of mosquitoes in the peri-domiciliary environment [14].

Opportune diagnosis and treatment, especially if provided within 48 h of symptoms onset, is thought to have a relatively higher impact on *P. falciparum* transmission, as gametocytes for this species are usually not detected at patent levels before 5 days of the onset of symptoms, as compared to *P. vivax,* for which it occurs much earlier [18, 19]. In this study, most patients were diagnosed within three days since the beginning of symptoms (94.3 %), demonstrating a sensitive and effective case detection system, which is likely to have played an important part on the striking reduction of cases observed in the period. After malaria diagnosis, treatment was immediately prescribed and given free of charge for all patients following the recommendations of the Brazilian official guidelines, including systematically a course of primaquine 0.5 mg/kg/day for 7 days for vivax malaria [12]. Unfortunately, treatment was not supervised, but patients were followed by an active surveillance of febrile cases, further suggesting a good compliance. Timely diagnosis and treatment, in parallel with and increase in the coverage of malaria vector control measures are suggested to be responsable by the successful reduction in malaria incidence.

Besides the later gametocytemia, hypnozoites responsible for the occurrence of relapses are also believed to be an important specific feature of *P. vivax* that contributes to this species burden and less notable responsiveness to the usual control measures [18]. This is more difficult to measure as recurring episodes can arise from new infections, recrudescence or relapses and there are no tools that can reliably discriminate between these conditions impairing more robust conclusions. In the lack of a design and tools specifically able to measure relapses, we have relied on the evaluation of recurrences to estimate the contribution of relapses to the burden of disease in the area. Although with marked variation, there was a trend towards greater contribution of recurring episodes to the total burden of *P. vivax* with reducing number of cases, which in some months accounted for up to 50 % of episodes, irrespective of bed net use and routine primaquine prescription. It has been shown through elegant investigations that relapses may account for up to 80 % of vivax malaria episodes in Papua New Guinea [20], where primaquine is not routinely administered, a feature that was also observed in other places [21, 22]. Our results suggest a high likelihood of relapses contributing to the burden of disease and would be in overall accordance to the expected interval for its occurrence in the region [23]. Our analyses demonstrated that having experienced a *P. vivax* episode (mono or mixed-infection) implied a considerable higher risk of presenting a new *P. vivax* clinical episode. Interestingly, for the specific analyses of recurrence, bed net use was not associated with protection, what would be consistent with the suggestion of a significant proportion being caused by relapses.

Interestingly, individuals were significantly more likely to have recurrent *P. vivax* infection after a monoinfection due to *P. falciparum*, in agreement with previous observations from Thailand [24]. The patients evaluated in Thailand and in our work did not receive hypnozoitocidal therapy for falciparum malaria and, if hypnozoites are present, relapse may occur [25]. One potential explanation for this phenomenon is that *P. falciparum* infection, especially in high density, is a proxy marker of malaria naivety and hence poor immunity to both *P. falciparum* and *P. vivax* infections. If this is true, relapses due to *P. vivax* hypnozoites acquired at or around the same time as the index *P. falciparum* infection would have a greater chance of reaching patency [24]. Historical records suggest that systemic parasitic and bacterial infections, but not viral infections, can activate *P. vivax* hypnozoites [26].

In non-endemic areas in Brazil, inadequate primaquine dosing was found to be associated with *P. vivax* malaria relapses [8, 9]. Unfortunately, patients adherence to anti-malarial treatment was not measured in the area nor performed specific pharmacological studies [27], as well as pharmacogenetic mutations cannot be discarded, such as CYP2D6, altering primaquine metabolism [28], factors that are well-known to alter the effectiveness of anti-relapse treatment. One hypothesis is that with a reduction of transmission intensity relapses may contribute to a relatively higher burden of disease, requiring the development of specifically-tailored strategies in similar scenarios.

In the cross-sectional evaluations, a low frequency of fever (22.7 % in T0 to 0 % at T12, T18 and T24) was observed among individuals with patent *Plasmodium* infection. In a different rural Amazon location, researchers have reported that conventional microscopy missed 54 % of *Plasmodium* carriers, indicating a large proportion of low-density infections in low transmission areas [29]. These findings suggest that apparently healthy carriers of patent parasitaemias are not detected by the routine health system activities and may have an important participation to the infectious reservoir contributing to maintain malaria transmission in this setting. Unfortunately, an important limitation of this study is not to test this population by PCR and the probable number of asymptomatic patients carrying *Plasmodium* in the area is likely to be considerably higher than the reported here. Positive microscopy in asymptomatic *Plasmodium* carriers was observed previously in the Brazilian Amazon [30–33].

One important finding of this study is that the reducing incidence of malaria was accompanied by a dramatic decrease on the overall incidence of acute fever episodes of 80 % from the first to the third year of follow-up. During the same period, the proportion of malaria-attributed fever also decreased considerably, from around 70 % during the high season in the first year of study to no higher than 20 % in the third year of follow-up. These findings have important implication for the local health system and transmission control activities. This figure surely reflects a high proportion of non-diagnosed fevers, and the ability of health systems to identify the causes of fever is important for both adequate individual case management and for health surveillance, as demonstrated in other areas [34–38]. This could be improved with the implementation of a broader and more comprehensive primary health care (PHC) in the region. Second, the reducing incidence of fever and clinical malaria episode make people less likely to seek for care in health facilities that only provide malaria diagnosis and treatment as is the case of most units in the rural Amazon region, making it more difficult to identify and tackle the infectious reservoir, what has consensually been agreed as an essential strategy for the rapid and sustained achievement of elimination [39, 40]. Ease of access to PHC has been already shown to be an important factor for reducing the burden of malaria [41]. Likewise, in a scenario of reduced funding, the issue of sustainability of malaria diagnosis and control activities is of main importance and may only be achieved with integration with other health care actions including a well-implemented syndromic surveillance of cases. An evaluation of what occurred in the area during the 3 years following the interruption of this study demonstrates that there was a resurgence of *P. falciparum* and an increase of the incidence of *P. vivax* (Additional file 1: Figure S1) and that this was accompanied by an increase on the proportion of individuals being diagnosed with malaria with more than 48 h of symptoms onset (from 6 % in 2010 to 45 % in 2013), what demonstrates the instability and vulnerability of malaria control in the current scenario. After the study was finished, the staff of the malaria diagnosis unit decreased, likely contributing for a less effective active search of malaria cases and spraying and IRS activities. How to best adapt and integrate malaria-specific and other conditions health care and control activities is still a major challenge and an area for investigation.

## Conclusions

Data presented here represent an important contribution to the knowledge of the epidemiology of malaria transmission and vector control in agricultural settlements in the Amazon and could be useful to assist on the development and implementation of strategies to control and eliminate this infection in the region. Eliminating *P. vivax* is more challenging than tackling *P. falciparum*, and may require specific tools and strategies for its containment, especially regarding the prevention of relapses [18, 42]. A trend of recurring malaria episodes was observed, likely to derive from relapses, to increase its relative contribution to the burden of disease. Relapsing *P. vivax* phenotypes may be more widespread and more prevalent in the Amazon than currently thought. This is an issue that remains to be more adequately investigated using enhanced approaches that would comprise pragmatic intervention trials and molecular methods [20], still to be adequately applied in the region. The Brazilian NMCP has launched a plan for elimination for *P. falciparum* in late 2015. Whether focusing on this species is a more appropriate strategy than attempting to eliminate overall malaria is yet to be determined. In order to achieve a rapid and sustained elimination of malaria transmission in the region, health systems and control strategies will be required to adapt to changing epidemiological scenarios through integrated and innovative approaches for which design scientists, health authorities and communities will have to be working together towards its achievement.

## Additional files

10.1186/s12936-016-1326-2 Temporal variation of malaria incidence in the study area, according to official surveillance (SIVEP-Malaria), from March 2003 to March 2015.
10.1186/s12936-016-1326-2 Survival analysis showing the risk of *Plasmodium vivax* recurrence according to bed net use.
