# Supplementary figures and images for: Declining malaria transmission in rural Amazon: changing epidemiology and challenges to achieve elimination
Source: Malar J. 2016 May 10;15:266. doi: 10.1186/s12936-016-1326-2 (PMC4863332; doi:10.1186/s12936-016-1326-2)

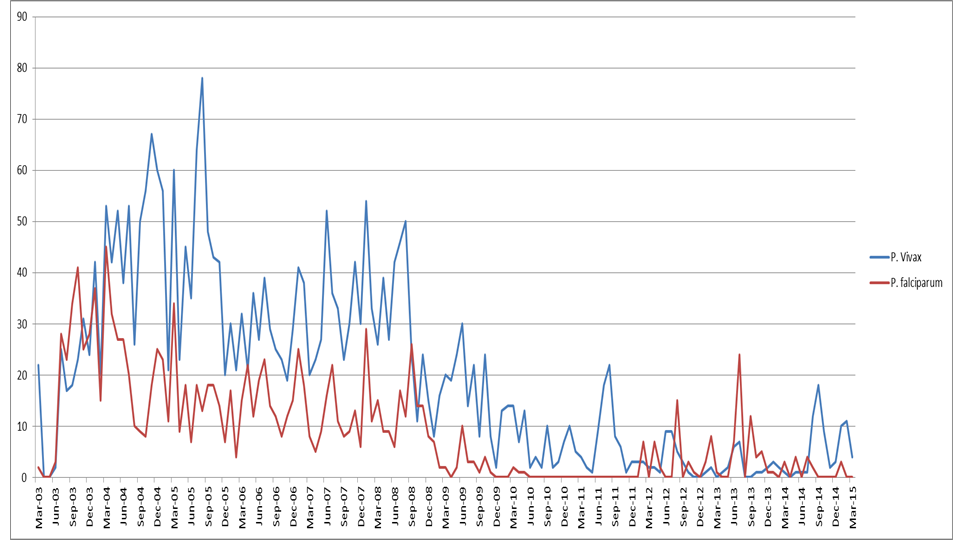

Supplement: Supplementary file 1 — 10.1186/s12936-016-1326-2 Temporal variation of malaria incidence in the study area, according to official surveillance (SIVEP-Malaria), from March 2003 to March 2015. [file 12936_2016_1326_MOESM1_ESM.tif]

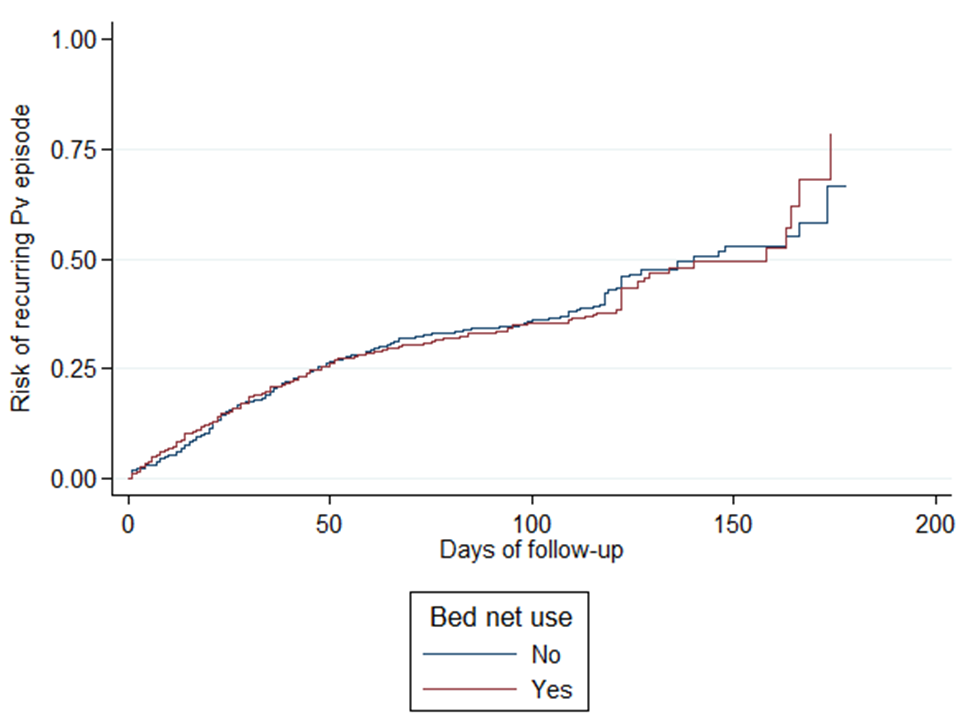

Supplement: Supplementary file 2 — 10.1186/s12936-016-1326-2 Survival analysis showing the risk of Plasmodium vivax recurrence according to bed net use. [file 12936_2016_1326_MOESM2_ESM.tif]
